# Improving care for women with obstetric fistula: new WHO recommendation on duration of bladder catheterisation after the surgical repair of a simple obstetric urinary fistula

**DOI:** 10.1111/1471-0528.15276

**Published:** 2018-06-06

**Authors:** M Widmer, Ö Tunçalp, MR Torloni, OT Oladapo, M Bucagu, AM Gülmezoglu

**Affiliations:** ^1^ UNDP‐UNFPA‐UNICEF‐WHO‐World Bank Special Programme of Research, Development and Research Training in Human Reproduction (HRP) World Health Organization Geneva Switzerland; ^2^ Department of Medicine Evidence Based Health Care Post‐Graduate Program São Paulo Federal University São Paulo SP Brazil; ^3^ Department of Maternal, Newborn, Child and Adolescent Health World Health Organization Geneva Switzerland

Under the Sustainable Development Goals and universal health coverage, the ‘survive, thrive, transform’ agenda moves beyond reducing mortality and focuses on the importance of maternal morbidity.^1^ An obstetric fistula, one of the most devastating types of maternal morbidity, is usually caused by injury during childbirth from prolonged or obstructed labour. The prolonged compression of the fetal head against the pelvic bones can cause ischaemic necrosis of parts of the bladder, urethra or vagina, resulting in an abnormal opening between a woman's genital tract and her urinary tract that leads to the continuous flow of urine through the vagina.^2^ Women with obstetric urinary fistula are often faced with serious social problems including abandonment by their partners, families and communities mainly due to persistent odour of urine as they are constantly wet and unable to control their urinary function.^3^ Although these fistulae are almost non‐existent in high‐income countries, it remains a public health problem that affects over one million women, their families and communities in Sub‐Saharan Africa and South Asia with poorly resourced health systems and inadequate intrapartum care services.^2^

Most obstetric urinary fistulae can be repaired surgically. Although most surgeons agree that continuous urine drainage is important to allow tension‐free healing of the surgical scar, opinions vary as to the length of time that a bladder catheter should be left in situ. In clinical practice, the duration of routine postoperative bladder catheterisation ranges from 5 to 42 days and has direct health, quality of life and cost implications.^4^ Long bladder catheterisation is associated with more discomfort for women, increased risk of infection and erosion related to the persistent contact of the catheter, need for longer stay at the hospital to receive intensive nursing care (women cannot be managed as outpatients due to their catheter needs) and therefore higher cost per patient.^5^

On 11 January 2018, the World Health Organization (WHO) released new guidance on duration of bladder catheterisation after the surgical repair of simple obstetric urinary fistula.^6^ In line with WHO guideline development processes,^7^ a systematic review was conducted to identify, critically appraise and synthesise the data on duration of bladder catheterisation after the surgical repair of simple obstetric urinary fistula^8^ and identified only two randomised trials involving a total of approximately 700 women. Both studies were open label, controlled, non‐inferiority, randomised trials conducted in eight African countries. The systematic review concluded that shorter (up to 10 days) duration of bladder catheterisation after the surgical repair of a simple obstetric urinary fistula is not associated with significant differences in outcomes, when compared with longer duration (> 10 days) of catheterisation, the critical outcomes being fistula repair breakdown before and after hospital discharge, extended hospital stay, urinary incontinence after discharge and maternal satisfaction with care. The overall certainty of evidence (based on the GRADE approach^9^) was graded as low.

WHO convened a panel of experts to review the results of the systematic review and to assess the balance between benefits and harms, values and preferences and resource implications of shorter versus longer duration of catheterisation. The panel recognised that there are several ways of defining the severity of fistula. For the purposes of this recommendation, a ‘simple’ fistula was considered as a mid‐anterior vaginal wall fistula with minimal scarring and with a diameter of 3 cm or less. The panel acknowledged the variation in the use of bladder catheter after fistula surgery and noted that some surgeons may consider a short duration of catheterisation as <7 days. However, for the purpose of the recommendation, the panel defined short duration as 7–10 days. The panel of experts accepted the uncertainty in the outcomes for shorter and longer duration of bladder catheterisation in light of other benefits such as improvement of women's comfort, potential reduction in the risk of infections associated with the catheterisation, and decrease in the woman's need for healthcare services. Finally, it was noted that although shorter hospitalisation associated with shorter postoperative bladder catheterisation would increase the availability of fistula care services (to potentially more women), this should be carefully balanced with the quality of services (i.e. the provision of a comprehensive care package to women recovering from obstetric fistula).

Based on this evidence, the panel concluded that for women in the postoperative period after the surgical repair of a simple obstetric urinary fistula, short duration bladder catheterisation (7–10 days) is recommended as an alternative to longer duration of catheterisation.

Women with obstetric fistulae reflect the failure of health systems to deliver accessible, timely and appropriate intrapartum care.^10^ There is a vital need to increase access to and strengthen the capacity of health facilities to provide high‐quality services for repair and care of women living with fistulae. We believe that the implementation of this WHO recommendation is a step in the right direction to improve the quality of care offered to women with obstetric urinary fistulae. Effective monitoring and evaluation based on the recommended set of indicators (fistula repair breakdown before hospital discharge, fistula repair breakdown after hospital discharge, urinary incontinence after hospital discharge, length of hospital stay and maternal satisfaction with care) will be crucial for the remaining research questions around even shorter duration of catheterisation (3–5 days), types of obstetric urinary fistula – including the ones that are not simple – and cost‐effectiveness.

As part of the efforts to offer universal health coverage, the dissemination and effective implementation and monitoring of this recommendation should be considered by countries and stakeholders involved in the provision of care for women with obstetric urinary fistulae at the global, national and local levels.

## Disclosure of interests

None declared. Completed disclosure of interest forms are available to view online as supporting information.

## Contribution to authorship

MW drafted the first version of the commentary. ÖT, MRT, OTO, MB and AMG provided feedback and edits, and approved the final version of the commentary.

## Details of ethics approval

Not required.

## Funding

Funding for this guideline by USAID was supplemented by UNDP‐UNFPA‐UNICEF‐WHO‐World Bank Special Programme of Research, Development and Research Training in Human Reproduction (HRP) core budget. The views of the funding bodies have not influenced the content of this guideline.
